# Sarcopenia Seems to Be Common in Older Patients With Restless Legs Syndrome

**DOI:** 10.1002/jcsm.13637

**Published:** 2024-11-20

**Authors:** Açelya Gokdeniz Yildirim, Derya Kaya, Fatma Sena Dost, Mehmet Selman Ontan, Ahmet Turan Isik

**Affiliations:** ^1^ Department of Geriatric Medicine, Faculty of Medicine Dokuz Eylul University Izmir Turkey; ^2^ Geriatric Sciences Association Izmir Turkey

**Keywords:** low muscle strength, restless legs syndrome, sarcopenia, slow gait speed

## Abstract

**Background:**

Restless legs syndrome (RLS) is a disorder characterized by nocturnally exacerbating pain that leads to significant sleep disturbances. The hormonal and metabolic changes caused by sleep disruption may increase the incidence of muscle‐related diseases like sarcopenia in older adults, which is defined by a progressive loss of muscle strength and mass. This study aimed to investigate the relationship between RLS and sarcopenia, which may affect each other through common pathophysiological pathways in older adults.

**Methods:**

This was a cross‐sectional study including 109 patients with RLS and 220 without RLS who applied to the geriatric clinic. RLS was assessed using the Turkish version of the International Restless Legs Syndrome Study Group (IRLSSG). Sarcopenia was diagnosed according to the European Working Group on Sarcopenia in Older People‐2 criteria. All the demographics, comorbid conditions, medications and findings of comprehensive geriatric assessments were recorded. The association between RLS and sarcopenia was examined by logistic regression.

**Results:**

The mean age was 75 ± 7.4 and 73.8 ± 7 years for the RLS and the control groups, respectively (*p* > 0.05) and the ratio of females was higher in the RLS group (69.7% vs. 57.9%) (*p* = 0.035). The frequencies of coronary artery disease (CAD), hypertension (HT) and peripheral artery disease (PAD) were significantly higher in RLS patients (*p* = 0.020, *p* = 0.047, *p* = 0.010, respectively), while the prevalence of anaemia was 41% and 25‐OH Vitamin D levels (25[OH]D) were higher than in the control group (*p* < 0.001). The frequency of probable sarcopenia and sarcopenia was higher in patients with RLS than in controls (20% vs. 11%, *p* = 0.037 and 8% vs. 2.3%, *p* = 0.047, respectively). A significant association between RLS and an increased likelihood of probable sarcopenia, sarcopenia and slow gait speed (odds ratio [OR]: 2.621, 95% confidence interval [CI] [1.265, 5.431]; OR: 4.542, 95% CI [1.284, 16.071]; OR: 2.663, 95% CI [1.432, 4.951], respectively) was found after adjusting for factors such as gender, HT, CAD, PAD, serum 25(OH)D levels, anaemia, chronic kidney disease (CKD) and nutritional status. However, the significance of low muscle mass disappeared (*p* > 0.05).

**Conclusion:**

This study demonstrated that sarcopenia is prevalent among older patients with RLS, which seems to be associated with low muscle strength and slow gait speed. Given the negative health outcomes related to sarcopenia, interventions aimed at preventing its development could be significantly beneficial for patients with RLS in older adults as well.

## 1 Introduction

Restless legs syndrome (RLS) is a neurological disorder characterized by a nocturnally exacerbated sensation of restlessness in the legs, which leads to significant sleep disturbances. This sensation is primarily experienced at night during periods of rest and causes an uncontrollable urge to move the legs, often due to discomfort. Although RLS is a common condition among younger populations, a late‐onset variant of RLS is also prevalent and underestimated in older adults [1]. Its prevalence varies between studies, but is as high as 35% in older adults [2]. RLS is a chronic neurological disorder with a prolonged prognosis. The severe leg pain frequently experienced by patients has an adverse effect on muscle well‐being and results in muscular inflammation [3]. Pain‐induced peripheral hypoxia is a significant factor contributing to this inflammation [4]. Peripheral hypoxia results in insufficient oxygen delivery to the muscle and inadequate elimination of toxins. Furthermore, sleep disruption, which is a sign of RLS, contributes to an increase in inflammation and metabolic inefficiency, resulting in a decrease in muscle mass and functionality [5]. Sleep difficulties also result in alterations in anabolic hormones, such as cortisol and Igf‐1, which accelerate the breakdown of proteins and have a negative impact on muscle health [6]. Moreover, a lack of physical activity caused by intense discomfort might result in muscular atrophy [7]. All these aforementioned changes raise the question that RLS may be related to sarcopenia in older adults.

Sarcopenia, one of the most common geriatric syndromes, is characterized by an age‐related decline in muscle mass, strength and functionality. The prevalence is 13%–14% under the age of 70 years while it is over 50% over the age of 80 years [8]. An average loss of 0.25 kg/year of lean muscle mass has been reported after the age of 22 years in terms of sarcopenia [9]. In fact, sarcopenia, decline in muscle mass, strength and functionality are age‐related health declining conditions. Sarcopenia is a progressive disease and is associated with many negative health outcomes, such as an increased risk of falls, dependency, morbidity and mortality among older adults [10, 11, 12]. In particular, medical costs resulting from falls and increased dependency are a medico‐socioeconomic problem. Therefore, it is crucial to evaluate coincident exhausting conditions, such as RLS, in patients with sarcopenia. Moreover, improving muscle strength and sarcopenia with interventions such as aerobic and resistance exercises in patients with RLS may reduce the severity of RLS symptoms and may decrease the negative effects of RLS, such as the risk of falling and sleep disorders [5, 7].

Recent studies have considered that RLS and sarcopenia may share common pathophysiological mechanisms, such as peripheral hypoxia and inflammation [13, 14]. It has been proposed that sleep disturbance‐related alterations in anabolic hormones [6], along with oxidative stress and inflammatory processes seen in patients with RLS, might also contribute to the development of sarcopenia [15, 16]. These mechanisms lead to muscle atrophy, especially in type 2 fibres associated with sarcopenia [3]. On the contrary, the increased oxidative stress, inflammation and sympathetic activity associated with sarcopenia may exacerbate symptoms of RLS [17]. All the common mechanisms make us think of a bidirectional relationship between both conditions in older adults. However, the relationship between RLS and sarcopenia has not been thoroughly explored in older adults so far. This study aimed to evaluate the potential relationship between RLS and sarcopenia in older adults.

## 2 Material and Methods

### 2.1 Participants

This cross‐sectional study included patients who attended to our geriatrics outpatient clinic in Dokuz Eylul University Hospital between November 2013 and September 2023. A total of 329 aged 60 and older (109 patients diagnosed with RLS and a total of 220 older adults as controls) were included in the study.

In the assessment of RLS, the Turkish version of the five criteria suggested by the International Restless Legs Syndrome Study Group (IRLSSG) was used: (1) an urge to move the legs, usually accompanied by uncomfortable and unpleasant sensations in the legs, sometimes the arms or other body parts are involved in addition to the legs; (2) the urge to move or unpleasant sensations begin or worsen during periods of rest or inactivity such as lying or sitting; (3) the urge to move or unpleasant sensations are partially or totally relieved by movement, such as walking or stretching; (4) the urge to move or unpleasant sensations are worse in the evening or night than during the day or only occur in the evening or night; (5) an urge to move the legs and accompanying unpleasant sensations that were not solely accounted for as symptoms primary to another medical or behavioural condition (e.g., myalgia, venous stasis, leg oedema, arthritis, leg cramps, positional discomfort and habitual foot tapping). Only those who were found to fulfil all of these five criteria were classified as having RLS. The IRLSSG rating scale (IRLS) was used to assess the severity of RLS. In this scale, the scores between 0 and 10 are considered mild, 11–20 as moderate, 21–30 as severe and > 30 as very severe [18]. The scale was employed to evaluate the severity of the disease in subjects diagnosed with RLS [19, 20].

Subjects who attended to the outpatient clinic for prevention programmes due to forgetfulness or other medical problems and had no history of movement disorders, dementia or psychiatric disorders, showed no symptoms or complaints of RLS and had undergone comprehensive geriatric assessments (CGA) as well as sarcopenia evaluations were included as the control group.

The exclusion criteria encompassed patients with acute conditions such as sepsis, adrenal insufficiency and delirium, those under the age of 60 years, individuals with a history of alcohol and substance abuse and those diagnosed with bipolar or psychotic disorders.

### 2.2 CGA and Laboratory Parameters

In this study, patient demographics including age, gender, educational background, comorbid conditions, history of falls and duration of diabetes were meticulously documented. For the CGA, several evaluations were conducted: the Barthel Index (BI) and Lawton and Brody Instrumental Activities of Daily Living (IADL) Scale assessed daily living activities; and the Mini Nutritional Assessment (MNA) was used for nutritional evaluation. Patients who scored 11 or lower on the MNA received an extended MNA assessment. Those scoring below 23.5 points were categorized as malnourished. The Tinetti test was employed for balance and gait analysis [11].

The laboratory tests including estimated glomerular filtration rate (eGFR), serum 25(OH)D, Thyroid‐Stimulating Hormone (TSH), magnesium, ferritin and haemoglobin levels were recorded. Males with haemoglobin levels below 13 g/dL and females with levels below 12 g/dL were classified as anaemic, and patients with eGFR < 60 were defined as having CKD. These tests were performed using an autoanalyser Diagnostic Modular System (E170 and P‐800 Diagnostic Modular System; Roche, Basel, Switzerland). 25(OH)D levels were quantified through radioimmunoassay.

### 2.3 Sarcopenia Assessment

The diagnosis of sarcopenia in patients adhered to the criteria established by the European Working Group on Sarcopenia in Older People (EWGSOP‐2) [10]. Muscle strength was evaluated using the Jamar hand dynamometer. During this test, patients were advised to maintain their elbow at a 90° flexion, keep the forearm in a neutral position and the wrist between 0 to 30° of extension. They were instructed to squeeze the dynamometer in this posture for 5 s, repeating the action three times, with the average of these results calculated. In these measurements, grip strength below 14 kg for women and below 28 kg for men indicated low hand strength. A gait speed below 0.8 m/s was considered indicative of reduced walking ability in the 4‐m gait test. Bioelectrical impedance analysis (BIA) was conducted using the MC‐780 U Multi Frequency Segmental Body Composition Analyzer (Tanita, Tokyo, Japan). The analysis employed a conversion equation calibrated against lean mass reference values measured by dual‐energy X‐ray absorptiometry in a specific population, defined as (kg) = (height (cm)^2^/R × 0.401) + (gender × 3.825) + (age × − 0.071) + 5.102. The skeletal muscle mass index (SMI) was calculated by dividing the obtained result by the square of the patient's height in meters. Threshold values for SMI were set at 8.33 kg/m^2^ for men and 5.7 kg/m^2^ for women [21]. The classification of sarcopenia was based on a hierarchy of criteria: probable sarcopenia was indicated by low muscle strength and sarcopenia by both low muscle strength and muscle mass [10].

### 2.4 Statistical Analysis

Categorical variables were expressed as numbers and percentages (*n*; %) while continuous variables were presented as mean ± standard deviation and median [minimum–maximum] values. The interpretation of categorical variables was conducted using *χ* ^2^ tests. The Kolmogorov–Smirnov test was employed to assess the normality of the distribution of continuous variables. The Student's *t*‐test was used to compare the means of two independent groups of normally distributed continuous variables while the Mann–Whitney *U* test was used to compare medians in cases where the variables were not normally distributed. In the analyses, RLS was the dependent variable, and probable sarcopenia, sarcopenia, low muscle mass and slow gait speed were the independent variables. Binary logistic regression analysis was used for the factors that were likely to affect probable sarcopenia, sarcopenia, slow gait speed and low muscle mass in patients with RLS. Adjusted models were established according to confounders detected between the groups as gender (model 1), gender, HT, CAD and PAD (model 2), gender, HT, CAD, PAD, 25(OH)D, anaemia, CKD and malnutrition (model 3). Correlation analyses between IRLS and sarcopenia parameters were performed using Pearson and Spearman analysis. A *p* value of < 0.05 was considered significant.

## 3 Results

This study was conducted with 109 RLS patients and the 220 controls who were similar means of ages (75 ± 7.4 years vs. 73.8 ± 7.2 years, respectively; *p* > 0.05) and years of education (7.5 ± 4.6 years vs. 8.4 ± 5.4 years respectively; *p* > 0.05). The percentage of female individuals were more than in the controls (69.7% vs. 57.9%; *p* = 0.035). The majority of RLS patients (52.5%) reported severe symptoms, and the median score of all RLS patients was 21 (5–40) on the IRLS. The prevalence of comorbid conditions such as CAD, HT and PAD was significantly higher in the RLS group, (27.8% for CAD, 72.2% for HT and 8.4% for PAD (*p* = 0.020, *p* = 0.047, *p* = 0.010, respectively). In contrast, the frequencies of diabetes mellitus (DM), cerebrovascular disease (CVD) and CKD were similar between the groups (*p* > 0.05), as was the duration of DM (20 ± 9.8 for RLS patients vs. 14.5 ± 9.5 for control group, respectively). Furthermore, there were no significant differences in the use of medications such as selective serotonin reuptake inhibitors, serotonin and norepinephrine reuptake inhibitors, dipeptidyl peptidase‐4 inhibitors, angiotensin‐converting enzyme inhibitors and angiotensin receptor blockers (*p* > 0.05). The ratio of patients with anaemia and mean levels of Vitamin D in the RLS group were higher than in the controls (*p* < 0.001 for each comparison). A 4‐m gait speed was reported to be slower in RLS patients (*p* = 0.010). Grip strength (kg) measurements were lower in the RLS group in both male and female (*p* = 0.034, *p* = 0.001, respectively) while SMI (kg/m^2^) measurements were similar (*p* > 0.05). All these patients' characteristics were summarized in Table 1.

**TABLE 1:** Patients characteristics.

	RLS(+)*n* = 109	RLS(−)*n* = 220	*p*
Demographic characteristics			
Age	75 ± 7.4	73.8 ± 7.2	*p* = 0.160
Sex (F%)	69.7%	57.9%	*p =* 0.035*
Education years	7.5 ± 4.6	8.4 ± 5.4	*p* = 0.205
RLS* score	21 (5–40)	—	—
Comorbidities			
CAD*	27.8%	16.7%	*p =* 0.020*
DM*	39.4%	33.3%	*p* = 0.274
HT*	72.2%	61.1%	*p =* 0.047*
PAD*	8.4%	2.3%	*p =* 0.010*
CVD*	5.6%	4.5%	*p* = 0.676
CKD*	27.2%	18.6%	*p* = 0.081
Medications			
SSRI*	23.1%	21.7%	*p* = 0.770
SNRI*	13.9%	9.5%	*p* = 0.226
DPP4*	13.9%	8.1%	*p* = 0.103
ACEI*	17.6%	13.6%	*p* = 0.345
ARB*	38.9%	33.5%	*p* = 0.335
Laboratory parameters			
Anaemia (F < 12 g/dL; M < 13 g/dL)	41%	27.3%	*p <* 0.001*
Magnesium	0.8 ± 0.2	0.8 ± 0.1	*p* = 0.646
TSH*	1.47 (0.05–8.6)	1.71 (0.01–13.1)	*p* = 0.409
25(OH)D*	28.5 ± 35.6	20.9 ± 13.1	*p <* 0.001*
Ferritin	50.1 ± 53.1	58.5 ± 66.1	*p* = 0.415
Sarcopenia‐related parameters			
4‐m gait speed (m/s)	0.77 (0. 12‐1.85)	0.84 (0. 2‐2)	*p =* 0.010*
Grip strength (kg) mean (min–max)			
Female	17.43 (3‐40)	21.28 (10‐45)	*p =* 0.001*
Male	28.76 (11‐42)	34.26 (2‐55)	*p =* 0.034*
SMI* (kg/m^2^) Mean (min–max)			
Female	8.01 (4.64–11.93)	8.07 (3.58–11.82)	*p* = 0.536
Male	9.8 (5.47–12.16)	10.08 (5. 46‐13.89)	*p* = 0.613

*Note:* Anaemia was defined as haemoglobin levels below 13 g/dL in males and 12 g/dL in females.

Abbreviations: 25(OH)D, 25‐hydroxy vitamin D; ACEI, angiotensin‐converting enzyme inhibitors; ARB, angiotensin receptor blockers; CAD, coronary artery disease; CKD, chronic kidney disease; CVD, cerebrovascular disease; DM, diabetes mellitus; DPP4, dipeptidyl peptidase‐4 inhibitors; HT, hypertension; PAD, peripheral artery disease; RLS, restless leg syndrome; SMI, skeletal muscle mass index; SNRI, serotonin‐norepinephrine reuptake inhibitors; SSRI, selective serotonin reuptake inhibitor; TSH, thyroid‐stimulating hormone.

* *p* < 0.05 statistically significant.

**FIGURE 1:**
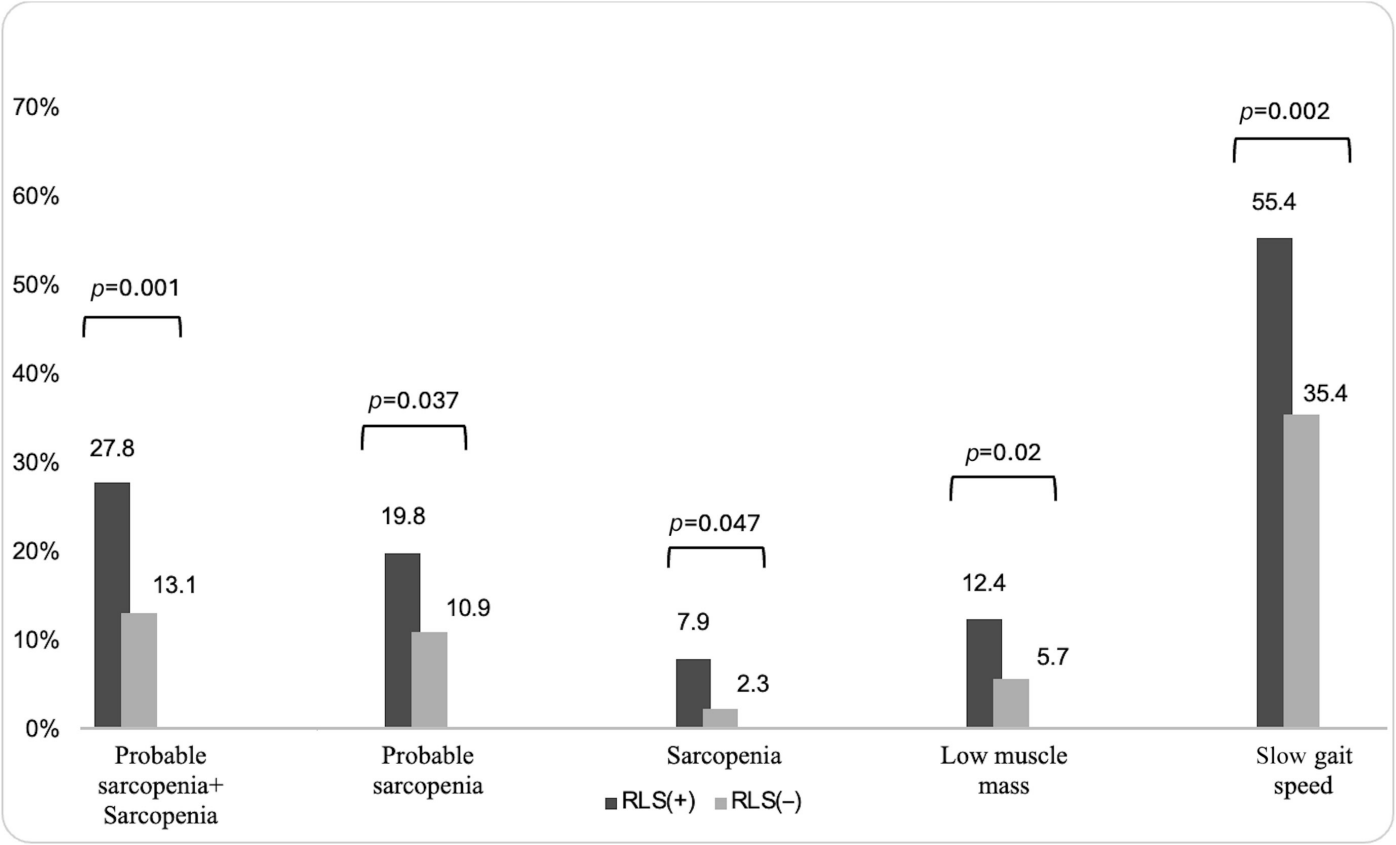
The frequency of probable sarcopenia, sarcopenia, low muscle mass and slow gait speed in all patients. *p* < 0.05, statistically significant.

The prevalence of geriatric syndromes such as probable sarcopenia and sarcopenia was significantly higher in the RLS group, at 19.8% and 7.9%, respectively (*p* = 0.037, *p* = 0.047) (Figure 1). The group also exhibited slow gait speeds and low muscle mass (*p* = 0.001, *p* = 0.02, respectively) and lower scores in balance and gait as per the Tinetti test (*p* = 0.001). Furthermore, RLS patients were more likely have malnutrition than the control group (*p* = 0.005) (Table 2).

**TABLE 2:** Clinical characteristics of patients according to comprehensive geriatric assessment.

		RLS (+) *n* = 109	RLS (−) *n* = 220	*p*
Malnutrition		28.0%	14.3%	*p* = 0.005 *
POMA		26 (0–28)	28 (1–28)	*p* = 0.001 *
BADL		95 (5–100)	95(10‐100)	*p =* 0.036 *
IADL		20(1‐23)	21(0–23)	*p* = 0.542

Abbreviations: BADL, basic activities of daily living (Barthel Index); IADL, Instrumental Activities of Daily Living (Lawton and Brody); POMA, Performance‐Oriented Mobility Assessment; RLS, restless leg syndrome.

* *p* < 0.05 statistically significant.

A regression analysis that adjusted for factors such as gender, HT, CAD, PAD, 25(OH)D levels, anaemia, CKD and nutritional status indicated a significant association between RLS and an increased likelihood of probable sarcopenia, sarcopenia and slow gait speed (odds ratio [OR]: 2.621, 95% confidence interval [CI] [1.265, 5.431]; OR: 4.542, 95% CI [1.284, 16.071]; OR: 2.663, 95% CI [1.432, 4.951], respectively). Although low muscle mass was significantly associated with RLS first and second models, this relationship did not persist in model 3 (*p* > 0.05) (Tables 3 and S5).

**TABLE 3:** The relationship between RLS and sarcopenia.

Restless legs syndrome	OR	95% CI	*p*
Model 1^a^			
Probable sarcopenia	2.799	1.505–5.208	*p* = 0.001*
Sarcopenia	4.518	1.348–15.139	*p* = 0.015*
Slow gait speed	2.279	1.378–3.768	*p* = 0.001*
Low muscle mass	2.946	1.204–7.206	*p* = 0.018*
Model 2^b^			
Probable sarcopenia	2.452	1.298–4.632	*p* = 0.006*
Sarcopenia	4.644	1.370–15.740	*p* = 0.014*
Slow gait speed	2.045	1.222–3.421	*p* = 0.006*
Low muscle mass	3.588	1.433–8.984	*p* = 0.006*
Model 3^c^			
Probable sarcopenia	2.610	1.259–5.409	*p* = 0.010*
Sarcopenia	4.542	1.284–16.071	*p* = 0.019*
Slow gait speed	2.663	1.432–4.951	*p* = 0.002*
Low muscle mass	2.322	0.787–6.852	*p* = 0.127

*Note:* References no: gender: female; HT: none; CAD: none; PAD: none; Anaemia: none; CKD: none; Malnutrition: none.

Abbreviations: CAD, coronary artery disease; CI, confidence interval; CKD, chronic kidney disease; HT, hypertension; OR, odds ratio; PAD, peripheral artery disease; 25(OH)D, 25‐Hydroxy Vitamin D.

^a^ Model 1: Regression analysis was adjusted on gender.

^b^ Model 2: Regression analysis was adjusted on gender, HT, CAD and PAD.

^c^ Model 3: Regression analysis was adjusted on gender, HT, CAD, PAD, 25(OH)D, anaemia, CKD and malnutrition.

* *p* < 0.05 statistically significant.

When gender differences in patients with RLS were evaluated, the frequency of female patients was higher in RLS group (69.7%, *n* = 76, *p* = 0.035). The demographic variables regarding female and male patients with RLS were shown in Tables S1 and S2. Female patients with RLS were more likely to have probable sarcopenia, sarcopenia, low muscle mass and slower gait speed than the female control group (Table S3). Also a high frequency of RLS in individuals with probable sarcopenia, sarcopenia, slow gait speed and low muscle mass was detected (Table S4).

**FIGURE 2:**
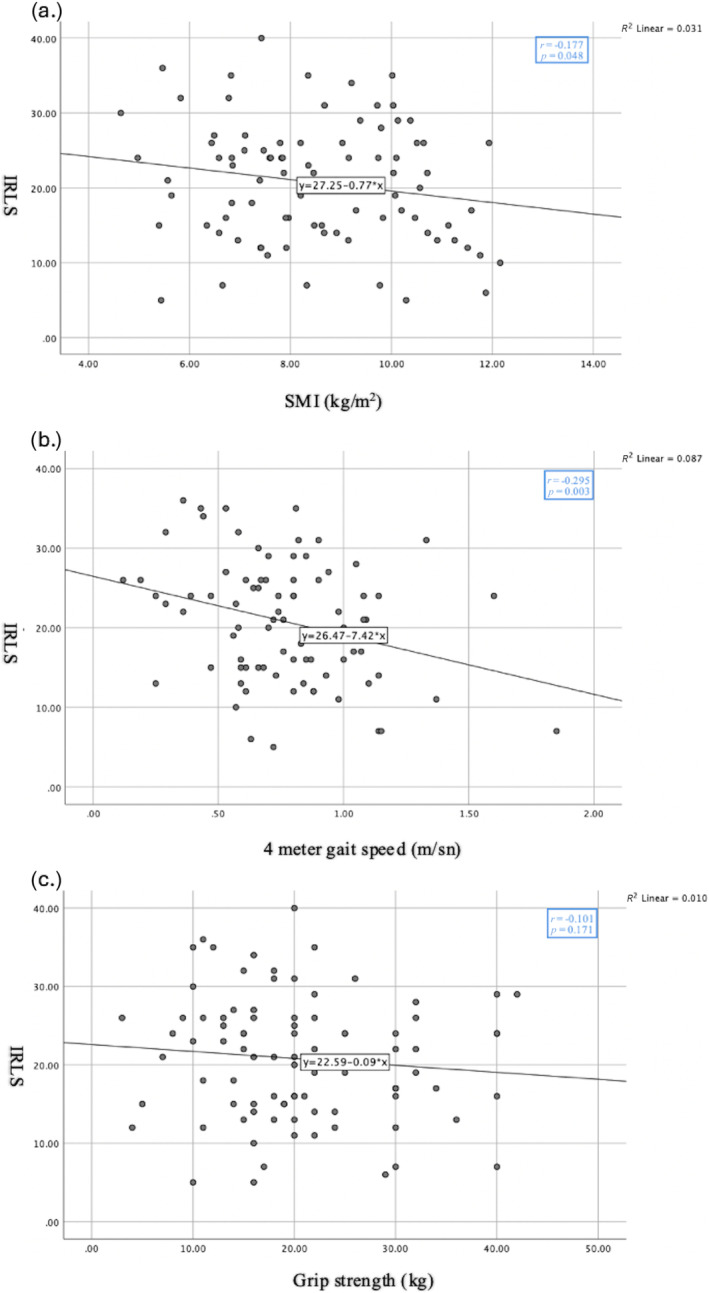
Correlation analysis between IRLS (IRLSSG rating scale) and sarcopenia parameters. a. Correlation analysis between IRLS and SMI (The Skeletal Muscle Mass Index) (kg/m^2^), r:Correlation Coefficient. b. Correlation analysis between IRLS and 4‐m gait speed (m/sn), r:Correlation Coefficient. c. Correlation analysis between IRLS and grip strength (kg), r:Correlation Coefficient. *p* < 0.05, statistically significant.

In the correlation analysis, SMI and 4‐m gait speed were negatively correlated with the IRLS (*p* = 0.048, *p* = 0.003, respectively) while no correlation was observed with grip strength (*p* = 0.171) (Figure 2).

## 4 Discussion

This cross‐sectional study demonstrated that sarcopenia is common in older adults with RLS and that low muscle strength and slow gait speed are independently related to this condition.

Recent studies have reported that RLS may be linked to muscle atrophy through various mechanisms, which have not been thoroughly evaluated to date. The current study aimed to clarify this issue and found that the frequency of probable sarcopenia and sarcopenia was higher in patients with RLS than in controls (20% vs. 11% and 8% vs. 2.3%, respectively). This increase may be associated with the low muscle strength and slow gait speed observed in patients with RLS. In these patients, the prevalence of low muscle strength (probable sarcopenia or dynapenia) and slow gait speed increased both by 2.6 times (*p* < 0.010 and *p* < 0.002, respectively) while the prevalence of sarcopenia increased by 4.5 times (*p* < 0.019). It is important to note that the study design does not allow for the establishment of a cause‐and‐effect relationship between the two conditions. However, when explaining the observed association, it is essential to consider the following potential explanations: (i) RLS may cause sarcopenia; (ii) sarcopenia may cause RLS; and/or (iii) a third factor may play a role in the development of both RLS and sarcopenia.

Initially, it is imperative to identify the contributory factors associated with the development of sarcopenia in patients diagnosed with RLS. These include peripheral hypoxia, the sedentary behaviour patterns of patients or inadequate or non‐restorative sleep. It has been demonstrated that hypoxia in peripheral tissues resulting from microvascular abnormalities [4] may increase micro vessel tortuosity in both slow‐twitch type I and fast‐twitch type II muscle fibres [3] and upregulate vascular endothelial growth factor [22] in the skeletal muscles of patients with RLS. Microvascular abnormalities may be more severe in the RLS group due to cardiovascular diseases such as CAD, HT and PAD. Although the underlying mechanisms are unclear, they may be related to atherosclerosis and microarousals that cause repeated increases in blood pressure and heart rate throughout the night [23, 24]. For instance, extremity ischaemia in patients with PAD leads to overproduction of oxidative stress, mitochondrial disorders and increased inflammation which are the pathophysiology of sarcopenia, in muscle tissue [25]. Additionally, anaemia and RLS are two conditions that have been shown to be associated in the literature. Iron deficiency is quite common in patients with RLS (around 25%) [26], and the prevalence of RLS in patients with iron deficiency anaemia is between 25% and 35% [27]. Within this perspective, the higher frequency of anaemia (41%) in our patients with RLS strengthens the peripheral hypoxia hypothesis. There are also publications showing that iron treatment reduces RLS symptoms [26]. However, the pathophysiology of the disease is caused by local iron deficiency in the brain rather than systemic one, suggesting that anaemia, independent of ferritin (storage iron), may be a risk factor leading to peripheral hypoxia in the increase of RLS symptoms [28, 29]. In brief, the presence of anaemia may lead to a compensatory increase in heart rate and blood pressure to ensure oxygen transport, affecting microvascular circulation. Given the fact that these microvascular changes may be linked to the severity of RLS symptoms and the majority of our patients presented with severe to very severe symptoms, it is unsurprising that the hypoxia in the muscles of RLS patients may be related to sarcopenia. Nevertheless, our findings suggest that RLS was independently associated with sarcopenia, even after adjusting for all these factors such as anaemia and comorbid conditions.

Furthermore, the fact that the majority of patients with RLS are sedentary [30, 31], as RLS symptoms may limit their physical activity, could induce the occurrence of sarcopenia [7]. Accordingly, the majority of our RLS patients exhibited reduced muscle strength and slow gait speed which suggests a sedentary lifestyle. Additionally, due to the lack of movement, continuous increases in pain and inflammation in RLS patients may cause sarcopenia, adversely affecting the muscles. Consequently, these two conditions may interact in a mutually reinforcing manner, leading to the development of sarcopenia. Furthermore, the well‐documented disturbances in sleep quality and quantity in RLS may also contribute to sarcopenia. Non‐restorative sleep has been linked to muscle atrophy observed in RLS patients [32]. It is evident that chronic non‐restorative sleep drives metabolic dysfunction, directly affecting muscle metabolism, which is associated with the loss of muscle mass and function [5, 32]. Even a single night of total sleep deprivation is sufficient to induce anabolic resistance, resulting in a reduction in skeletal muscle protein synthesis rates by 18% and the creation of a pro‐catabolic environment, as evidenced by an increase in plasma cortisol and a reduction in plasma testosterone and insulin‐like growth factor (IGF‐1) levels [6, 33].

This metabolic damage, caused by lower testosterone and IGF‐1 and higher cortisol levels, may drive pathways involved in systemic inflammation, insulin resistance and may suppress muscle protein synthesis, leading to dysregulated skeletal muscle metabolism and energy balance. All these changes increase the risk of sarcopenia [6, 34, 35]. The reduction of testosterone and IGF‐1 levels with age [36] may render patients with RLS more vulnerable to sarcopenia in the geriatric population currently experiencing sleep disorders. In light of the aforementioned evidence, it can be reasonably postulated that RLS may induce sarcopenia.

Vitamin D has been reported to have important effects on skeletal muscle function, upregulating mitochondrial ATP production in skeletal muscle and reducing oxidative stress through overexpression of vitamin D receptors [37]. Furthermore, overexpression of receptors has been associated with skeletal muscle hypertrophy [38]. Our finding, the frequency of sarcopenia is higher in RLS patients with high 25(OH)D levels, needs further investigation by follow‐up studies.

Conversely, sarcopenia may also lead to RLS by several mechanisms. To begin with, neurological processes such as dopaminergic downregulation, inadequate motor programming, or a reduction in motor unit and a decreased motor unit firing rate [39, 40], which are related to the development of sarcopenia, or dynapenia may also result in RLS [41]. Furthermore, cytokines that play a role in inflammaging, including interleukin‐6 and tumours necrosis factor‐alpha, in conjunction with adipokines and myokines, may render the muscle more susceptible to developing sarcopenia [42, 43]. Chronic low‐grade inflammation in sarcopenic older adults may also mediate RLS. Recent studies have indicated that a high concentration of inflammatory markers is associated with RLS, thereby increasing the probability of experiencing frequent RLS symptoms [44]. As well as inflammation, increased oxidative stress and sympathetic activity associated with sarcopenia may also cause RLS symptoms [16]. In conclusion, recent studies have underscored the crucial roles of lifestyle factors, such as unhealthy nutrition [42], low physical activity, or prolonged sedentary time [45] and smoking [46], in the risk of developing sarcopenia. These lifestyle factors related to sarcopenia, including obesity, physical inactivity and smoking, have also been suggested to be associated with the risk of developing RLS in prospective cohort studies [30]. Physical inactivity, in particular, appears to create a vicious cycle in sarcopenia, leading to the speculation that it may worsen or cause RLS symptoms.

The study has several notable strengths. Sarcopenia was diagnosed in accordance with the revised EWGSOP‐2 criteria [10], and thresholds for low muscle strength and low muscle mass were defined based on the findings for Turkish older adults [11]. All patients underwent a CGA, which also included an evaluation of sarcopenia and the diagnostic questionnaire for RLS. To the best of our knowledge, this is the first study to evaluate sarcopenia in older patients with RLS. However, it should be noted that the present study is subject to certain limitations. These include its retrospective cross‐sectional design and the use of BIA, an indirect method, to measure muscle mass. Additionally, detailed data about sleep status of the patients were missing.

## 5 Conclusion

This study demonstrated that sarcopenia is prevalent among older patients with RLS, which seems to be associated with low muscle strength, sarcopenia and slow gait speed. Considering the sarcopenia‐related negative health outcomes in older adults, interventions to prevent the development of sarcopenia would also be of paramount importance for RLS patients and vice versa.

## Ethics Statement

Our study was approved by the Dokuz Eylul University Non‐Interventional Research Ethics Committee on 18 January 2021, under the decision number 2021/02‐40. The study was conducted in accordance with the ethical standards outlined in the 1964 Declaration of Helsinki and its subsequent amendments. Additionally, informed consent was obtained from all participants included in the study. The authors of this manuscript certify that they comply with the ethical guidelines for authorship and publishing in the *Journal of Cachexia, Sarcopenia and Muscle*.

## Conflicts of Interest

The authors declare no conflicts of interest.

## Supporting information


**Table S1** Characteristics of female patients

**Table S2** Characteristics of male patients

**Table S3** The frequencies of sarcopenia and related parameters in female patients with RLS

**Table S4** The frequencies of RLS in patients with Sarcopenia

**Table S5** The relationship between RLS and sarcopenia
