# Supplementary material for: Sarcopenia Seems to Be Common in Older Patients With Restless Legs Syndrome
Source: J Cachexia Sarcopenia Muscle. 2024 Nov 20;16(1):e13637. doi: 10.1002/jcsm.13637 (PMC11670161; doi:10.1002/jcsm.13637)
Supplement: Supplementary file 1 — Table S1 Characteristics of female patients [file JCSM-16-e13637-s005.docx]

**Table S1.** Characteristics of female patients

|  | **RLS (+) (n=76)** | **RLS (-) (n=127)** | **p value** |
| --- | --- | --- | --- |
| Age | 75.7 + 7.1 | 73.1 + 7.7 | *p=0.019* |
| **COMORBIDITIES** |  | | |
| HT* | %78.7 | %65.4 | *p=0.045* |
| CVD* | %4.1 | %3.9 | p=0.967 |
| PAD* | %9.5 | %0 | *p=0.001* |
| CAD* | %28 | %14.3 | *p=0.017* |
| DM* | %38.2 | %35.4 | p=0.696 |
| CKD* | %30.6 | %18.4 | p=0.05 |
| **MEDICATIONS** |  | | |
| SSRI* | %20 | %22.2 | p=0.710 |
| SNRI* | %14.7 | %9.4 | p=0.259 |
| ACEI* | %16 | %12.6 | p=0.499 |
| ARB* | %46.7 | %40.9 | p=0.428 |
| DPP4I* | %12 | %7.1 | p=0.236 |
| **LABORATORY PARAMETERS** |  | | |
| Anemia | %41.7 | %26.4 | *p=0.027* |
| Magnesium | 0.87 (0.5-2) | 0.82 (0.53-1.03) | p=0.059 |
| 25(OH)D* | 25.06 (4.2-58.5) | 20.97 (4.79-75) | p=0.057 |
| Ferritin | 47.95 (2.5-334) | 58.6 (4.1-414) | p=0.979 |
| TSH* | 1.9 (0.5-8.6) | 1.97 (0.01-13.1) | p=0.089 |
| Malnutrition | %23.1 | %13.3 | p=0.90 |

*HT: Hypertension, CVD: Cerebrovascular Disease, PAD: Peripheral Artery Disease, CAD: Coronary Artery Disease, DM: Diabetes Mellitus, CKD: Chronic kidney disease, SSRI: Selective Serotonin Reuptake Inhibitor, SNRI: Serotonin-Norepinephrine Reuptake Inhibitors, ACEI: Angiotensin-Converting Enzyme Inhibitors, ARB: Angiotensin Receptor Blockers, DPP4: Dipeptidyl Peptidase-4 Inhibitors, 25(OH)D: 25-Hydroxy Vitamin D, TSH: Thyroid Stimulating Hormon

p<0.05, statistically significant
