# Supplementary material for: Sarcopenia Seems to Be Common in Older Patients With Restless Legs Syndrome
Source: J Cachexia Sarcopenia Muscle. 2024 Nov 20;16(1):e13637. doi: 10.1002/jcsm.13637 (PMC11670161; doi:10.1002/jcsm.13637)
Supplement: Supplementary file 2 — Table S2 Characteristics of male patients [file JCSM-16-e13637-s003.docx]

**Table S2.** Characteristics of male patients

|  | **RLS (+) (n=33)** | **RLS (-) (n=93)** | **p value** |
| --- | --- | --- | --- |
| Age | 73.3 + 7.8 | 74.6+ 6.4 | p=0.344 |
| **COMORBIDITIES** |  | | |
| HT* | %57.6 | %54.8 | p=0.787 |
| CVD* | %9.1 | %5.4 | p=0.463 |
| PAD* | %6.1 | %5.4 | p=0.883 |
| CAD* | %27.3 | %19.4 | p=0.343 |
| DM* | %42.4 | %29 | p=0.160 |
| CKD* | %19.4 | %18.9 | p=0.955 |
| **MEDICATIONS** |  | | |
| SSRI* | %30.3 | %19.4 | p=0.195 |
| SNRI* | %12.1 | %9.7 | p=0.693 |
| ACEI* | %21.2 | %15.2 | p=0.431 |
| ARB* | %21.2 | %23.7 | p=0.775 |
| DPP4I* | %18.2 | %9.7 | p=0.197 |
| **LABORATORY PARAMETERS** |  | | |
| Anemia | %39.4 | %28.9 | p=0.269 |
| Magnesium | 0.84 (0.6-2) | 0.84 (0.53-1.03) | *p=0.028* |
| 25(OH)D* | 27.5 (7.3-54.6) | 19 (6.84-66.4) | *p>0.001* |
| Ferritin | 55.6 (10.6-238) | 76.7 (5-414) | p=0.368 |
| TSH* | 1.78 (0.55-5.28) | 1.76 (0.2-13.1) | p=0.461 |
| Malnutrition | %39.3 | %14.8 | *p=0.005* |

*HT: Hypertension, CVD: Cerebrovascular Disease, PAD: Peripheral Artery Disease, CAD: Coronary Artery Disease, DM: Diabetes Mellitus, CKD: Chronic kidney disease, SSRI: Selective Serotonin Reuptake Inhibitor, SNRI: Serotonin-Norepinephrine Reuptake Inhibitors, ACEI: Angiotensin-Converting Enzyme Inhibitors, ARB: Angiotensin Receptor Blockers, DPP4: Dipeptidyl Peptidase-4 Inhibitors, 25(OH)D: 25-Hydroxy Vitamin D, TSH: Thyroid Stimulating Hormon.

p<0.05, statistically significant
