# Supplementary material for: Sarcopenia Seems to Be Common in Older Patients With Restless Legs Syndrome
Source: J Cachexia Sarcopenia Muscle. 2024 Nov 20;16(1):e13637. doi: 10.1002/jcsm.13637 (PMC11670161; doi:10.1002/jcsm.13637)
Supplement: Supplementary file 3 — Table S3 The frequencies of sarcopenia and related parameters in female patients with RLS [file JCSM-16-e13637-s001.docx]

**Table S3.** The frequencies of sarcopenia and related parameters in female patients with RLS

|  | **Female** |  |  | **Male** |  |  |
| --- | --- | --- | --- | --- | --- | --- |
|  | **RLS (+) (%)** | **RLS (-) (%)** | **p value** | **RLS (+) (%)** | **RLS (-) (%)** | **p value** |
| Probable sarcopenia | %26.2 | %9.4 | *p=0.002* | %32 | %18.3 | p=0.136 |
| Sarcopenia | %6.2 | %0.8 | *p=0.046* | %12.5 | %4.3 | p=0.131 |
| Slow gait speed | %59.1 | %35.5 | *p=0.002* | %46.2 | %33.7 | p=0.249 |
| Low muscle mass | %9.2 | %2.5 | *p=0.039* | %20.8 | %10.1 | p=0.157 |

*RLS: Restless leg syndrome

p<0.05, statistically significant
