# Supplementary material for: Sarcopenia Seems to Be Common in Older Patients With Restless Legs Syndrome
Source: J Cachexia Sarcopenia Muscle. 2024 Nov 20;16(1):e13637. doi: 10.1002/jcsm.13637 (PMC11670161; doi:10.1002/jcsm.13637)
Supplement: Supplementary file 4 — Table S4 The frequencies of RLS in patients with Sarcopenia [file JCSM-16-e13637-s004.docx]

**Table S4.** The frequencies of RLS in patients with Sarcopenia

|  | Probable sarcopenia | | | Sarcopenia | | Slow gait speed | | Low muscle mass | |
| --- | --- | --- | --- | --- | --- | --- | --- | --- | --- |
|  | | (+) | (-) | (+) | (-) | (+) | (-) | (+) | (-) |
| RLS* (%) | | %46.3 | %25.4 | %58.3 | %27.6 | %41.1 | %23 | %47.8 | %28.2 |
| p value | | *p=0.002* | | *p=0.044* | | *p=0.001* | | *p=0.047* | |

*RLS: Restless leg syndrome

p<0.05, statistically significant
