# Supplementary material for: Sarcopenia Seems to Be Common in Older Patients With Restless Legs Syndrome
Source: J Cachexia Sarcopenia Muscle. 2024 Nov 20;16(1):e13637. doi: 10.1002/jcsm.13637 (PMC11670161; doi:10.1002/jcsm.13637)
Supplement: Supplementary file 5 — Table S5 The relationship between RLS and sarcopenia [file JCSM-16-e13637-s002.docx]

**Table S5.** The relationship between RLS and sarcopenia

| Restless Legs Syndrome | OR | | %95 CI | p value |
| --- | --- | --- | --- | --- |
| **Model 1** |  |  | |  |
| Probable Sarcopenia | *2.799* | 1.505-5.208 | | *p=0.001* |
| **Gender** | ***0.477*** | **0.275-0.826** | | ***p=0.008*** |
| Sarcopenia | *4.518* | 1.348-15.139 | | *p=0.015* |
| **Gender** | ***0.466*** | **0.268-0.810** | | ***p=0.007*** |
| Slow Gait Speed | *2.279* | 1.378-3.768 | | *p=0.001* |
| **Gender** | ***-*** | **-** | | **p=0.057** |
| Low Muscle Mass | *2.946* | 1.204-7.206 | | *p=0.018* |
| **Gender** | ***0.453*** | **0.258-0.793** | | ***p=0.006*** |
| **Model 2** |  |  | |  |
| Probable Sarcopenia | *2.452* | 1.298-4.632 | | *p=0.006* |
| **Gender** | ***0.473*** | **0.269-0.834** | | ***p=0.010*** |
| **HT** | ***-*** | **-** | | **p=0.344** |
| **CAD** | ***-*** | **-** | | **p=0.309** |
| **PAD** | ***-*** | **-** | | **p=0.119** |
| Sarcopenia | *4.644* | 1.370-15.740 | | *p=0.014* |
| **Gender** | ***0.452*** | **0.255-0.802** | | ***p=0.007*** |
| **HT** | ***-*** | **-** | | **p=0.309** |
| **CAD** | ***-*** | **-** | | **p=0.116** |
| **PAD** | ***-*** | **-** | | **p=0.099** |
| Slow Gait Speed | *2.045* | 1.222-3.421 | | *p=0.006* |
| **Gender** | ***-*** | **-** | | ***p=0.057*** |
| **HT** | ***-*** | **-** | | **p=0.396** |
| **CAD** | ***-*** | **-** | | **p=0.417** |
| **PAD** | ***-*** | **-** | | **p=0.145** |
| Low Muscle Mass | *3.588* | 1.433-8.984 | | *p=0.006* |
| **Gender** | ***0.422*** | **0.235-0.756** | | ***p=0.004*** |
| **HT** | ***-*** | **-** | | **p=0.288** |
| **CAD** | ***-*** | **-** | | **p=0.056** |
| **PAD** | ***-*** | **-** | | **p=0.100** |
| **Model 3** |  |  | |  |
| Probable Sarcopenia | *2.610* | 1.259-5.409 | | *p=0.010* |
| **Gender** | ***0.474*** | **0.250-0.899** | | ***p=0.022*** |
| **HT** | ***-*** | **-** | | **p=0.205** |
| **CAD** | ***-*** | **-** | | **p=0.857** |
| **PAD** | ***-*** | **-** | | **p=0.087** |
| **25(OH)D** | ***1.033*** | **1.010-1.056** | | ***p=0.005*** |
| **Anemia** | ***-*** | **-** | | **p=0.157** |
| **CKD** | ***-*** | **-** | | **p=0.733** |
| **Malnutrition** | ***-*** | **-** | | **p=0.166** |
| Sarcopenia | *4.542* | 1.284-16.071 | | *p=0.019* |
| **Gender** | ***0.463*** | **0.243-0.884** | | ***p=0.020*** |
| ***HT** | ***-*** | **-** | | **p=0.228** |
| ***CAD** | ***-*** | **-** | | **p=0.544** |
| ***PAD** | ***-*** | **-** | | **p=0.063** |
| ***25(OH)D** | ***1.028*** | **1.006-1.051** | | ***p=0.012*** |
| **Anemia** | ***-*** | **-** | | **p=0.120** |
| ***CKD** | ***-*** | **-** | | **p=0.542** |
| **Malnutrition** | ***-*** | **-** | | **p=0.177** |
| Slow Gait Speed | *2.663* | 1.432-4.951 | | *p=0.002* |
| **Gender** | ***-*** | **-** | | **p=0.092** |
| **HT** | ***-*** | **-** | | **p=0.382** |
| **CAD** | ***-*** | **-** | | **p=0.845** |
| **PAD** | ***-*** | **-** | | **p=0.114** |
| **25(OH)D** | ***1.034*** | **1.011-1.057** | | ***p=0.004*** |
| **Anemia** | ***-*** | **-** | | **p=0.126** |
| **CKD** | ***-*** | **-** | | **p=0.745** |
| **Malnutrition** | ***-*** | **-** | | **p=0.172** |
| Low Muscle Mass | 2.322 | 0.787-6.852 | | p=0.127 |
| **Gender** | ***0.468*** | **0.246-0.891** | | ***p=0.021*** |
| **HT** | ***-*** | **-** | | **p=0.177** |
| **CAD** | ***-*** | **-** | | **p=0.414** |
| **PAD** | ***-*** | **-** | | **p=0.068** |
| **25(OH)D** | ***1.027*** | **1.005-1.049** | | ***p=0.017*** |
| **Anemia** | ***-*** | **-** | | **p=0.087** |
| **CKD** | ***-*** | **-** | | **p=0.459** |
| **Malnutrition** | ***-*** | **-** | | **p=0.268** |

*OR: Odds Ratio, CI:Confidence Interval, **HT: Hypertension, CAD: Coronary Artery Disease, PAD: Peripheral Artery Disease, 25(OH)D: 25-Hydroxy Vitamin D, CKD: Chronic kidney disease**

**References no: Gender: female, HT: none, CAD: none, PAD: none, Anemia: none, CKD: none, Malnutrition: none**

Model-1 Regression analysis was adjusted on gender.

Model-2 Regression analysis was adjusted on gender, HT, CAD, and PAD.

Model-3 Regression analysis was adjusted on gender, HT, CAD, PAD, 25(OH)D, anemia, CKD and malnutrition.

p<0.05, statistically significant
